# What do family physicians consider an error? A comparison of definitions and physician perception

**DOI:** 10.1186/1471-2296-7-73

**Published:** 2006-12-08

**Authors:** Nancy C Elder, Harini Pallerla, Saundra Regan

**Affiliations:** 1 Department of Family Medicine, University of Cincinnati, Cincinnati, Ohio, 45267-0582, USA

## Abstract

**Background:**

Physicians are being asked to report errors from primary care, but little is known about how they apply the term "error." This study qualitatively assesses the relationship between the variety of error definitions found in the medical literature and physicians' assessments of whether an error occurred in a series of clinical scenarios.

**Methods:**

A systematic literature review and pilot survey results were analyzed qualitatively to search for insights into what may affect the use of the term error. The National Library of Medicine was systematically searched for medical error definitions. Survey participants were a random sample of active members of the American Academy of Family Physicians (AAFP) and a selected sample of family physician patient safety "experts." A survey consisting of 5 clinical scenarios with problems (wrong test performed, abnormal result not followed-up, abnormal result overlooked, blood tube broken and missing scan results) was sent by mail to AAFP members and by e-mail to the experts. Physicians were asked to judge if an error occurred. A qualitative analysis was performed via "immersion and crystallization" of emergent insights from the collected data.

**Results:**

While one definition, that originated by James Reason, predominated the literature search, we found 25 different definitions for error in the medical literature. Surveys were returned by 28.5% of 1000 AAFP members and 92% of 25 experts. Of the 5 scenarios, 100% felt overlooking an abnormal result was an error. For other scenarios there was less agreement (experts and AAFP members, respectively agreeing an error occurred): 100 and 87% when the wrong test was performed, 96 and 87% when an abnormal test was not followed up, 74 and 62% when scan results were not available during a patient visit, and 57 and 47% when a blood tube was broken. Through qualitative analysis, we found that three areas may affect how physicians make decisions about error: the process that occurred vs. the outcome that occurred, rare vs. common occurrences and system vs. individual responsibility

**Conclusion:**

There is a lack of consensus about what constitutes an error both in the medical literature and in decision making by family physicians. These potential areas of confusion need further study.

## Background

"Language exerts hidden power, like a moon on the tides." (Rita Mae Brown, Starting From Scratch, New York: Bantam, 1988)

What we call things matters – reports of medical errors "channel attention, shape interpretations and serve as springboards for action"[1]. Many entities require the reporting of errors, [2-7] and with the passage of the United States Patient Safety and Quality Improvement Act of 2005, it is likely that even more physicians will be asked to identify and report errors[8]. Once a domain primarily of hospitals, the importance of medical errors occurring in the outpatient, primary care setting has become more apparent, [9-15] and physicians are being asked to report errors from this venue as well.

Yet, while physicians are being asked to find, report and reduce medical errors in their practices, we lack a universally understood definition of exactly what is meant by "medical error"[1,16-19]. Previous primary care studies have demonstrated large differences in the number of errors reported by individual physicians, even within the same practices[12,20] and research in hospitals has found differences in how health care workers interpret terms like error, incident and event[21,22]. Individual interpretation of what is an error plays a role in identifying errors and making reports[23,24]. While collations of some patient safety terms from the literature have been done, [25,26] how these definitions affect physicians' use of terms like "error" is not clear.

In order to better understand what may affect a physician's understanding of "medical error," we performed a three step process: 1) We systematically collected definitions for medical error found in the medical literature; 2) We surveyed family physicians and family physician medical error "experts" about whether they felt a medical error occurred in a series of common clinical scenarios; and 3) We qualitatively explored both the definitions and the survey findings to see if a model of factors might help explain how physicians make decisions about whether to call something an error.

## Methods

### Literature search

Using the MESH term Medical Error/classification, we retrieved 216 English language articles from 1985 – October, 2005. All abstracts were reviewed, and 68 articles dealing with "medical error" or "error" were reviewed for definitions. Further articles containing definitions were gleaned from the medical errors literature searching with the MESH term Medical Error and the secondary text phrases "primary care," "family medicine" and "error reporting" (93, 37 and 78 abstracts reviewed, and a total of 16 additional articles reviewed). Definitions that focused exclusively on a subset of error, such as medication or diagnosis, were excluded. In addition, the report of the World Health Organization on a draft comparative glossary of patient safety terms, prepared by the Joint Commission for the Accreditation of Healthcare Organizations was also reviewed[25,27]. In addition, definitions used in national surveys (United States) and polls were collated and added to the definition list.

### Survey development

From the literature[2,10,12] and our experience, we devised 5 common scenarios that were representative of preventable problems experienced by family physicians. Due to the frequency and importance of the testing process in primary care, [10,12,28,29] we chose this area in which to concentrate our scenarios, as they were likely to be familiar and real to practicing physicians. These scenarios were then sent to a group of primary care medical error researchers for review and a draft of the survey was pilot tested on 10 local family physicians for refinement prior to the final survey. The final five scenarios are found in table 1. We purposely chose to include scenarios that included variability around a number of factors, including medical conditions, systems and individuals in a practice and knowledge and severity of clinical outcomes. In asking our participants their opinions on the scenarios, we asked whether "an error or mistake had occurred." We used both words because some definitions state that an error is a plan not achieving its goal while a mistake is an incorrect plan,[30] and we wanted to be inclusive of both issues. We did not, however, define either term on the survey, as our intention was to assess agreement about their use as free standing words.

**Table 1:** Clinical scenarios used in the survey

Name	Scenario
Mr. Black/LFT	Dr. Jones ordered liver function tests to evaluate Mr. Black's health complaints. The next day, a report of Mr. Black's lipids (but not liver tests) shows up on Dr. Jones' desk and they are normal. Dr. Jones documents "normal lipids, notify patient" and sends it to his nurse. A week later, Mr. Black returns, more ill, and is found to have acute hepatitis A.
Mrs. Rose/glucose	Mrs. Rose, a patient with high blood pressure, has a basic metabolic profile performed, and is found to have a random blood glucose of 189. Dr. Smith documents "have patient return for repeat glucose and glycohemoglobin." The nurse documents "attempted phone call, no answer." Eight months later, the patient returns with a yeast infection and is found to have a random blood glucose of 356
Ms Brown/TSH	Dr. Miller reviewed a large number of lab results from his "normal lab results" folder and sent them to be filed. The next month, he sees Ms Brown again for menstrual irregularities. In reviewing her chart, Dr. Miller sees he wrote "normal, file" next to an elevated TSH of 37.
Mr. White/broken tube	Mr. White got his blood drawn by Dr. Jones' medical assistant for an ordered test. After he left, she dropped the tube and broke it. Mr. White is called, and makes another visit to the office to get his blood drawn the next day.
Ms Green/CT results	Ms Green wants to know the results of head CT scan ordered by her doctor to evaluate her headaches. The test was done at the hospital X-ray department last week. She calls the office and leaves a message asking the doctor or nurse to call her. When no one returns her call, she calls back two days later and makes an appointment. At the visit, the CT results are not in her chart, and cannot be found in the office.

### Data collection

We elected to survey both a random sample of active family physician members of the AAFP as well as a selected sample of family physician "experts" that have presented, published or advocated nationally about medical error. We did this in order to look for a possible disconnect between the application of error by these two groups. After approval by our institutional review board, we mailed our survey and a cover letter to a random sample of 1000 active family physician members of the AAFP and by e-mail to 25 family physician experts. All non-responders were sent a second survey 3 weeks later. Demographic and practice data were also collected about each AAFP respondent. Data were entered into an excel database.

### Data analysis

Data were reviewed for accuracy, and descriptive statistics were performed using SPSS. The survey was performed for descriptive purposes only, and was not powered for statistical analyses. While the survey did not ask for comments, several participants added hand written comments and these were separated and reviewed. Data sources for the qualitative analysis were collated and included the survey scenarios, the survey results, hand written comments, and the definitions from the literature review. These were analyzed together using the qualitative technique of immersion and crystallization[31,32]. With this technique, we immersed ourselves in these data, gaining emergent insights. Immersion included reading, re-reading, organizing phrases and segments of written text, model building and discussion. We then participated in a series of discussions in order to explicate theories and synthesize ideas. We then returned to the medical literature and the data sources looking for both corroborating findings and alternative interpretations[31].

## Results

### Literature review

The most common definition found in the medical literature is that attributed to James Reason and published by the Institute of Medicine (IOM) in To Err is Human, "Failure of a planned action to be completed as intended or use of a wrong plan to achieve an aim"[33]. (Table 2) Because it is so commonly used, we included several variations on it. Interestingly, James Reason originally defined "error" only as the first part – failure of a planned action to be completed as intended. He used the term "mistake" for use of a wrong plan to achieve an aim[30]. However, the IOM report used "error" for both components, defining one as an error of execution and the other as an error of planning[33]. Reason also defined slips as relating to observable actions, associated with attentional failures and lapses as more internal and related to failures of memory[30].

**Table 2:** Medical error definitions from the medical literature

**Categories**	**Definition**
James Reason's definition	The failure of planned actions to achieve their desired goal. [55]
Based on James Reason's definition.	Failure of a planned action to be completed as intended or use of a wrong plan to achieve an aim; the accumulation of errors results in accidents. [33]
	The failure of a planned action to be completed as intended (i.e., error execution) or the use of a wrong plan to achieve an aim (i.e., error of planning). [58]
	The failure of a planned action to be completed as intended or the use of a wrong plan to achieve an aim. Errors can include problems in practice, products, procedures, and systems. [59]
From essays, editorials and reviews	An unintentional deviation from standard operating procedures or practice guidelines. [60]
	Deviation in a process of care that may or may not cause harm to patients. [61]
	An adverse event or near miss that is preventable with the current state of medical knowledge. [62]
	An act of commission or omission that substantively increases the risk of a medical adverse event. [63]
	A failure of a structure or process only to the extent that it prevents maximizing the outcomes of interest. [43]
	A failure to perform an intended action which was correct given the circumstances. It can only occur if there was or should have been an appropriate intention to act on the basis of a perceived or remembered state of events and if the action finally taken was not that which was or should have been intended. [64]
	Errors in healthcare are by definition, human errors, and human errors are errors in human actions. [65]
	Underlying causes of failed decisions for the failed delivery of care.... Errors are the causes of the failed processes, whether they are in decision making or in treatment delivery. [38]
	Failure to meet reasonable expectations for goal-directed activity. [42]
	Mistakes that encompass not only lapses in safety (mistakes in the provision of health care that expose patients to "additive" risk), but also include inattention to extant risks that patients bring to the encounter. [66]
	An act in the process of care that could harm a patient, therefore, measures of medical errors can be considered process measures. [19]
Used in research and reporting	An act of commission or omission that caused, or contributed to the cause of, the unintended injury. [49]
	Any event you don't wish to have happen again, that might represent a threat to patient safety. [48]
	Anything that happened in your own practice that should not have happened, that was not anticipated and that makes you say, "that should not happen in my practice and I don't want it to happen again. [10]
	A commission or omission with potentially negative consequences for the patient that would have been judged wrong by skilled and knowledgeable peers at the time it occurred, independent of whether there were any negative consequences [35]
	A failure to meet some realistic expectation (an action, process, diagnosis or endpoint). [41]
	An unintended event, no matter how seemingly trivial or commonplace, that could have harmed or did harm a patient. [9]
	An event that was not completed as intended and/or meant that work was disrupted in some way. [23]
Used in research and surveys with patients and the public	Sometimes when people are ill and receive medical care, mistakes are made that result in serious harm, such as death, disability or additional or prolonged treatment. These are called medical errors. [36]
	Some examples of medical mistakes are when a wrong dose of medicine is given, an operation is performed other than what was intended for the patient or results of a medical test are lost or overlooked [39]
	Preventable incidents that result in a perceived harm [14]

Of note is that several definitions include comments about actual or potential outcomes, for example, "a threat to patient safety,"[34] "potentially negative consequences,"[35] "result in a perceived harm,"[14] or "result in serious harm"[36]. Other definitions, however, focus only on the processes. In addition, some definitions focus on the human components,[37] while others more on systems[38]. Surveys and polls to both the public and physicians tend to define by example and include harmful outcomes in the definition[36,39].

### Survey

#### Participants

From the AAFP sample of active members, we received 284 usable surveys from 997 valid addresses for a 28.5% response rate. Demographic details of the respondents are found in Table 3. Our sample compares favorably with the total active membership of the AAFP. The AAFP is 32.3% women, and our sample was 35.8%. The age breakdown of the AAFP is almost identical to our age breakdown (30 – 44: AAFP 45.8%, our study 46%; 45–54: AAFP 45%, our study 48%; 60 and over: AAFP 6.8%, our study 6.0%). Our sample had 15% solo practitioners (18% in AAFP) and 25.2% in a multi-specialty practice (21.7% in AAFP)[40]. Twenty three of 25 "experts" responded to the survey (92%), and two-thirds of them were male with an average age of 47 (range 34 – 58).

**Table 3:** Demographics of participants

	AAFP	Experts
**Age range**	30 – 73 (ave. 48.3)	34 – 58 (ave.47)
**Gender**		
Male	64.2%	67%
Female	35.8%	33%
**Specialty scope of practice**		
Multi specialty	25.2%	
Family practice only	74.8%	
**Size of Practice**		
Solo	15.0%	
2 – 6	42.9%	
7 – 12	17.5%	
13 – 20	10.4%	
Greater than 20	14.3%	
**Residency practice location**		
Yes	27.7%	
No	72.3%	

#### Error occurrence

One hundred percent of the AAFP respondents believed an "error or mistake" occurred when a physician wrote "normal, file" on an abnormal result (Ms Brown/TSH), while only 47% felt that a dropped blood tube was an error (Mr. White/broken tube). (Table 4) Several physicians believed the broken blood tube was "an accident." Almost 14% of physicians felt they couldn't make a decision about whether an error occurred when confronted with the scenario about missing results (Ms Green/CT results), but 62% did believe there was an error. The experts' opinions mirrored those of the AAFP members. The experts committed to a decision in all the scenarios except the Ms Green/CT results scenario, with 13% being unable to decide if an error occurred.

**Table 4:** Percent of respondents who believed an error or mistake occurred in the described scenario.

Scenario	Yes, an error occurred		No, an error did not occur		Unable to tell if an error occurred	
	
	AAFP	Experts	AAFP	Experts	AAFP	Experts
Mr. Black/LFT	87%	100%	7%		6%	
Mrs. Rose/glc	87%	96%	9%	4%	4%	
Ms Brown/TSH	100%	100%				
Mr. White/broken tube	47%	57%	50%	43%	3%	
Ms Green/CT results	62%	74%	24%	13%	14%	13%

### Qualitative categorization of "error" decision making

Since all the scenarios described a situation where a "failure of a planned action to be completed as intended or use of a wrong plan to achieve an aim" occurred, we looked for additional explanatory processes to explain the variation in the survey results. We found three areas that we felt could explain physician decision making around "is this event is an error?": Do I know the outcome and is there harm from this event, is this event a common or a rare occurrence and does responsibility for this event lie predominantly with an individual or with the system? (Table 5) We found error definitions that imposed criteria for calling an event an error for all of these explanatory areas, and the findings of the survey supported their use in error decision making. Another area of potential conflict for physicians is that of "reasonable expectations"[10,41-43]. However, there were no survey scenarios, findings or comments that seemed to address this, and we chose not to include it in our model, but it does warrant further research.

**Table 5:** Factors associated with assigning error to a scenario as determined by qualitative analysis.

Error decision making factor	Survey questions and findings	Supporting definitions	Non-supporting definitions
Knowledge of harmful outcomes	87 – 100% agree an error occurred in scenarios where harm is most evident (clinical symptoms continue, worsen or develop) 13 – 14% unable to make a decision about error where outcome is most unknown (missing test result)	"increases the risk of medical adverse event," "could harm a patient," "caused or contributed to unintended injury," "could have harmed or did harm a patient."	"failure of a planned action to be completed as intended or the use of a wrong plan."
Everydayness of event	26% to 53% disagree an error occurred in scenarios most likely to occur in physicians' offices (broken tube, lost test results)	"a failure to meet some realistic expectation"	"no matter how seemingly trivial or commonplace"
Individual responsibility	100% agree an error occurred in the scenario with most clear individual responsibility (missed abnormal result)	"errors in healthcare are human errors," "an act of commission or omission."	"failed processes," "a failure of a structure or process."

**Figure 1:**
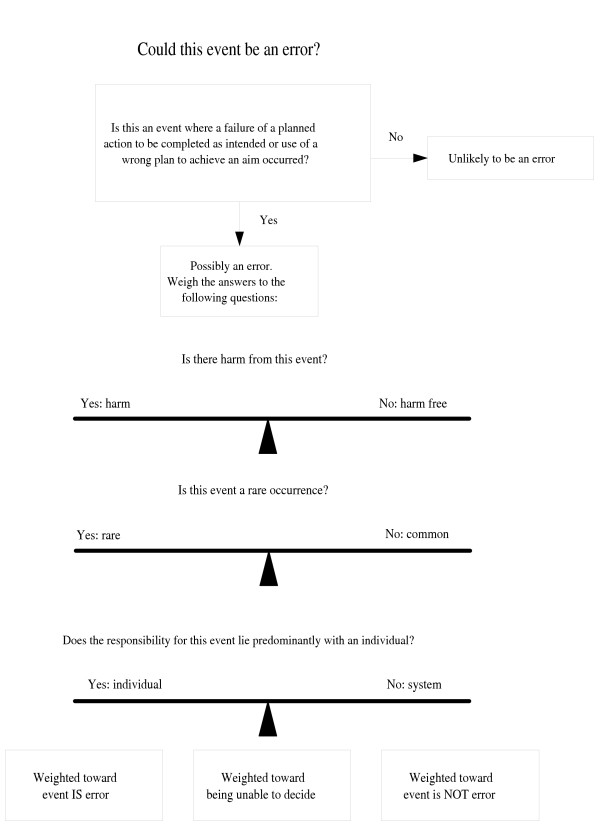
A model of physician decision making when assessing whether an event should be classified as an error.

A model of the three questions used by physicians in the decision making process is found in figure 1. As physicians ponder calling an event an error, they first make the decision that something went wrong, and then look to these three areas to tip the balance towards or away from calling the event an error. Using a survey scenario, for example, Ms Brown/TSH had harm, is likely a rare occurrence and has clear individual responsibility, and 100% of participants felt it was an error.

## Discussion

Error and its many synonyms not only appear in medical journals, they are words used frequently in daily conversation. James Reason's definition is widely accepted, frequently cited in the medical literature and encompasses human and system processes[33,44]. Yet, in attempts to clarify, expand and modify, many others have also defined medical error. (Table 2) After deciding something is "not right," additional questions, even if not explicitly asked, likely figure into the decision making process: Do I know the outcome and is there harm from this event, is this event a common or a rare occurrence and does responsibility for this event lie predominantly with an individual or with the system? (Figure 1)

In both the medical literature and for our participants, outcome seems to influence determinations about error[45,46]. Considering the outcome as well as the process in making decisions about error is not unusual[11,17]. As noted, several of the error definitions found in the literature include harm or potential harm in the definition[14,35,36,39,47-49]. Woods and Cook remark on this confusion by describing three ways in which "error" is used: error as the cause of failure (or poor outcome), as the failure itself or as departure from a standard process[46]. Hindsight and outcome bias describe how knowledge of the outcome affects the decision making about the quality of processes[50,51]. For example, if we can't find a test result when wanted, but it is normal and doesn't change our management, we define the lost result differently than when a result is abnormal, and now treatment has been significantly delayed[21]. The process of losing the result may be the same, but our "hindsight" of the "outcome" affects how we interpret that process. Our physician respondents may be so used to using the hindsight of outcome to assess a process, that without that information (especially with the missing CT result) it is difficult for them make a decision about just the process. Similar findings in other studies note that uncertain outcomes lead to larger proportions of respondents refusing to make a decision about error[21]. Tamuz and colleagues found that errors detected and corrected by staff in a hospital were felt to be non-events that occurred as a natural part of the work flow, and not error[1,22]. In the model in figure 1, knowledge of harm tips the balance towards labeling an event as error, but not knowing the outcome leaves the balance unswayed.

Some problems may occur so commonly in practice today, that it is difficult for physicians to perceive these as "errors." For example, there is probably not a practice in existence that draws blood that has not lost, broken or somehow damaged a blood specimen tube. And missing clinical information has recently been documented to occur in approximately 14% of all office visits[29]. The two scenarios we offered physicians of these commonly occurring events, Mr. White/broken tube and Ms Green/CT results, received the least agreement from physicians that an error occurred. Perhaps the fact that these experiences are weekly, if not daily occurrences for many makes it difficult for physicians to acknowledge these as "errors." While these scenarios certainly fit definitions of medical error, [2,10,48], these "academic" definitions may seem disconnected from practicing physicians who have emotional and personal responses to words such as error[22,42,52]. Events that are infrequent and unexpected are more likely to tip the balance toward error (figure 1), whereas those that are common and expected (like an occasional broken blood tube or missing test results) tip the balance away from error[22,24].

There may also be differences in how errors are perceived by physicians whether the problem appears to be in the system or due to an individual's action. While three scenarios had strong agreement that an error occurred (Mr. Black/LFT, Mrs. Rose/glc and Ms Brown/TSH), in only one, where the physician misreads an abnormal TSH is there 100% agreement from all the participants. Traditionally, when errors occurred, the standard response was to "blame and shame"[33,53]. A responsible person is most clearly identified in the Ms Brown/TSH scenario, and may be part of the reason why all physicians identified an error in this scenario. Physicians may more easily identify errors where the decision and action of an individual are at fault, rather than where the system fell down. A qualitative, hospital based study previously found that complex system errors were more likely to be called "practice variances" or "suboptimal outcomes" rather than error[21]. Reason and others have addressed this complexity of both system problems and human actions by describing "latent" errors (the underlying system) and "active" errors (the human actions) [30,44,54,55]. But the emotional aspects of feeling responsible may tip the balance toward deciding an event is an error more than an understanding of complex systems.

There are several limitations to this study. The literature search was systematic, but not exhaustive. Non English language articles were not reviewed, and the medical errors literature is too large to review the body of all articles to see if a definition of error is proposed. However, our review did reveal a broad spectrum of error definition, not previously collated and published. Our survey response rate of 28.5% from the AAFP solicitation is low, and certainly limits the generalizability of the results. Those who chose to respond may differ in their opinions about the issues under study. However, we used the survey qualitatively to illuminate and illustrate potential deficiencies in error definitions. The scenarios we devised came from our clinical experience and our experience in researching testing process errors in family physician offices[11,12,20]. Although the scenarios were reviewed for face validity and pilot tested, there still may have been some unclear sections of the scenarios, leading to responses that we might have misinterpreted. However, this method mimics the practical application of error decision making, and has been used successfully to study patient safety and errors[21,56,57]. Use of the surveys did not allow us to discover if factors such as knowledge of harm were being used inappropriately by our participants in making decisions about error, and this is an area for further research.

## Conclusion

Physicians are being asked to make reports of errors, and this is likely to increase in the future. Error reports can be essential to determining the focus of patient safety attention and interventions[1]. Further research is needed to better understand how physicians make decisions about calling an event an error. We generated a model from this study that proposes that three additional elements are important in making a decision about whether an event is an error: Do I know the outcome and is there harm from this event, is this event a common or a rare occurrence and does responsibility for this event lie predominantly with an individual or with the system? The relative importance of each of these areas, and their interrelationships need to be confirmed by further research, including both qualitative and quantitative studies. As we better understand how family physicians use the word "error" then the reports they make will be even more useful as springboards for action.

## Competing interests

The author(s) declare that they have no competing interests.

## Authors' contributions

NCE was the principal investigator, designed the study instruments, performed the literature search and led the qualitative analysis. HP and SR collected, entered and analyzed survey data. All authors participated in the qualitative analysis and the writing of the manuscript.

## Pre-publication history

The pre-publication history for this paper can be accessed here:


